# The Evolution of School Health and Nutrition in the Education Sector 2000–2015 in sub-Saharan Africa

**DOI:** 10.3389/fpubh.2016.00271

**Published:** 2017-01-30

**Authors:** Bachir Sarr, Meena Fernandes, Louise Banham, Donald Bundy, Amaya Gillespie, Brie McMahon, Francis Peel, K. C. Tang, Andy Tembon, Lesley Drake

**Affiliations:** ^1^Partnership for Child Development, Imperial College London, London, UK; ^2^Global Partnership for Education, Washington, DC, USA; ^3^Bill and Melinda Gates Foundation, Seattle, WA, USA; ^4^United Nations Children’s Fund, New York, NY, USA; ^5^Formerly World Health Organization, Geneva, Switzerland; ^6^World Bank, Washington, DC, USA

**Keywords:** school health and nutrition, policy analysis and decision making, education objectives, sub-Saharan Africa, Millennium Development Goals

## Abstract

**Study objectives:**

To document the progression of school health and nutrition and its integration within the education sector in sub-Saharan Africa between 2000 and 2015.

**Background:**

School health and nutrition programs have contributed to “Education for All” objectives by helping ensure that children benefit from quality education and reach their educational potential.

**Methods:**

Analysis of education sector plans (ESPs) in terms of the Focusing Resources on Effective School Health (FRESH) framework and the World Bank Systems Approach for Better Education Results (SABER) School Health survey from a set of countries in sub-Saharan Africa.

**Results:**

Between 2000 and 2015, the presence and scope of school health and nutrition as reflected in the four FRESH pillars grew substantially in ESPs. Three of these pillars have large, upfront costs. The fourth pillar requires recurring annual budgetary allotments.

**Conclusion:**

Governments clearly recognize that evidence-based, contextually designed school health and nutrition programs can contribute to education sector goals. Moving into the post-2015 era, these programs can also help draw the last 10% of children into school and enhance their readiness to learn.

## Introduction

Evidence shows that some of the most common health conditions of school-age children in low- and middle-income countries affect their access to education as well as learning outcomes (1). Such conditions include malaria, worm infections, hunger, anemia, tooth decay, diarrhea, and respiratory disease. Health and nutrition programs offered through the school platform can serve to prevent and treat these conditions, and disproportionately benefit the poor and vulnerable, who are more likely to suffer from ill health or poor nutrition. By leveraging the education system to deliver simple treatments for common conditions, school health and nutrition (SHN) programs can be highly cost-effective (2).

The recognition of SHN as a key component of education systems began in the 1980s when child mortality rates declined and the international health community began to shift focus to the development of the post-survival child. A body of evidence grew that demonstrated the need for a broad range of inputs from health, education, food, and community support, as well as demonstrating the potential of schools as an effective means of delivery. In the 1990s, the increased research across the sectors supported policy dialog and the development of policy frameworks, global alliances, and national school health policies. By the dawn of the new Millennium, SHN programs were becoming part of development policy worldwide (3).

The launch of a framework that aimed at Focusing Resources on Effective School Health (FRESH) at the World Education Forum in Dakar in 2000 was a landmark achievement in the recognition of the importance of SHN for the education sector (4). The organizations participating in this launch included UNESCO, UNICEF, WHO, and the World Bank. The Dakar Framework for Action to address Education for All (EFA) was also launched in 2000 and called on governments to develop education sector plans (ESPs) by 2002 to support the achievement of EFA goals and targets by the Dakar Framework (5). SHN was noted as a priority area in the Dakar Framework as a means to support the achievement of these goals and targets and continues to be reinforced. As exemplified by continuing recognition at the World Education Forum 2015 (6).

During the period of the Millennium Development Goals, great strides were made in getting more children into schools, with primary education enrollment rates in developing regions increasing from 83% in 2000 to 91% in 2015 (7). However, these gains were lower among the poor and vulnerable. For example, children from the poorest household are four times more likely to be out of school than those from high-income households (3, 7). Children with disabilities, from poor communities, orphaned by disease or from conflict areas are the least likely to attend school (8). School feeding can help address a key challenge looking forward which is to reach the last 10% of children who do not attend school and help ensure that they also have the opportunity to learn and reach their potential (8).

This retrospective analysis illustrates how these landmark activities – the launch of FRESH and the Dakar Framework – have contributed to the mainstreaming of SHN into the education sector in sub-Saharan African countries between 2000 and 2015. The study is a quantitative, cross-country assessment of this issue, and draws from two primary data sources – national ESPs and the school health survey sub-component of the World Bank Systems Approach for Better Education Results (SABER) School Health survey. Findings from the analysis provide insights into the process as well as identify remaining challenges in the new era of the sustainable development goals (SDGs).

## Materials and Methods

The investigation draws from data from two sources on national SHN programs. The first was ESPs from 25 countries in sub-Saharan Africa. The second source was SABER School Health surveys from 16 countries in the region. The analysis of data from each source is described in more detail below.

### Education Sector Plans

Education sector plans set country priorities deemed essential to meet their education objectives and highlight areas where funding is needed. In addition, they provide insight into how a country prioritizes school health and their key areas of concern. The plans are developed by governments in consultation with relevant stakeholders. A participatory process involving various sub-sectors of the ministries in charge of education, finance, labor, social development, parent teacher associations, and non-governmental organizations (NGOs) helps to ensure national ownership.

This study presents findings from ESPs from 25 countries that were developed since 2000, following the World Education Forum meeting in Dakar. The ESPs were obtained from Planipolis, an online portal for education plans and policies supported by the Global Partnership for Education. These countries were Benin, Burkina Faso, Burundi, Cameroon, Central African Republic, Chad, Eritrea, Ethiopia, Gambia, Ghana, Guinea Bissau, Guinea, Kenya, Liberia, Madagascar, Mali, Mauritania, Mozambique, Republic of Congo, Rwanda, Senegal, Togo, Uganda, Zambia, and Zimbabwe.

The ESPs were reviewed to identify SHN sub-components following the FRESH framework (4). FRESH was developed based on good practices in SHN programing and was launched at the World Education Forum in 2000 as a mechanism to support the development of effective school health policies, programs, and services. FRESH also underscored school health as a critical component toward the achievement of universal primary education. The framework includes four pillars: (1) health-related school policies, (2) safe learning environments, (3) skill-based health education, and (4) school-based health and nutrition services. Health-related school policies should be inclusive and gender sensitive, promoting the physical and psychosocial health not only of children but also teachers and other school staff. Safe learning environment refers to access to safe water and the provision of separate sanitation facilities for girls, boys, and teachers. Skill-based health education refers to the development of knowledge, attitudes, and skills that promote positive health behaviors. SHN services include low-cost, effective interventions such as the provision of deworming tablets, micronutrient supplements, or school meals.

The ESP review also identified SHN priorities through a search for the following keywords: rehabilitate, water, latrine, hygiene, health, screening, medical, school feeding, canteens, meals, deworming, and neglected tropical diseases. ESPs were coded in terms of the pillars represented as well as program activities.

### Systems Approach for Better Education Results (SABER)

The ESP analysis was complemented by an analysis of surveys from 16 sub-Saharan African countries using the SABER data collection tool. SABER is a policy gap analysis tool launched by the World Bank in 2011. The SABER program collects comparable data on the policies and institutions of education systems around the world and benchmarks them against good practice. SABER’s aim is to provide stakeholders with an objective, clear, and comprehensive snapshot of how well a country’s education system is oriented toward delivering learning. In this study, the SABER survey was used to look at the effectiveness and implementation of SHN policies. The survey was implemented in 16 African countries between 2011 and 2013. These countries were Benin, Cape Verde, Cote d’Ivoire, Ethiopia, Ghana, Kenya, Madagascar, Malawi, Mali, Niger, Nigeria, Rwanda, Senegal, Tanzania, Uganda, and Zanzibar.

## Results

**Figure 1:**
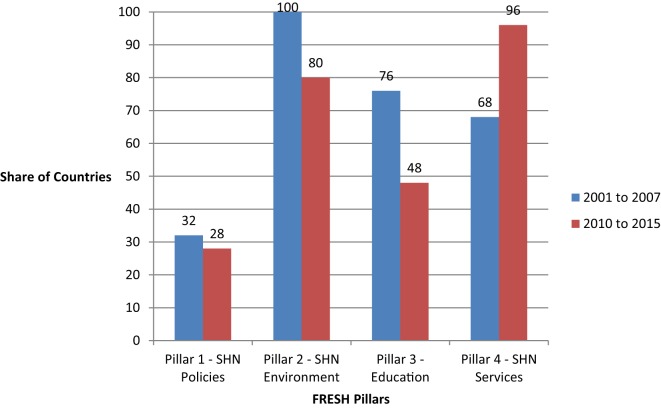
**Share of ESPs from sub-Saharan African Countries by FRESH Pillar**. Note: the ESPs from both the early and later periods were developed between 2000 and 2015. Early period refers to ESPs developed following the World Education Forum and the later period refers to the ESP developed subsequently.

Since 2000, SHN has been on the education sector agenda. Figure 1 illustrates the development of ESPs along the FRESH pillars over the period from the World Education Forum in 2000 to December 2015. The period was divided into two phases to investigate changes over time. For the set of countries, the early phase referred to ESPs developed directly following the World Education Forum, from 2001 to 2007. The later phase includes ESPs that were developed 10–15 years after the 2000 education forum.

Figure 1 shows that all ESPs reflected at least one of the FRESH pillar, already reflecting the recognition of the integral role of SHN in the education sector following the World Education Forum. Moreover, it reflects the funding needs for the different pillars. Pillars 1, 2, and 3 require large, upfront costs, while Pillar 4 requires recurring annual budgetary allotments.

For Pillar 1, the establishment of a health-based school policy typically requires significant, upfront investment. Most of the policies that countries sought to develop in the early period as presented in Figure 1 were focused on HIV, as a response to the pandemic underway on the continent which was threatening to reverse gains made in education and development during the previous years. By 2007, 79% of countries surveyed had an education sector-specific HIV/AIDS strategy with 83 and 80% of countries in the Eastern Africa and West Africa networks, respectively (9). Countries that sought to develop a school health policy in the later period were often different countries than whose which developed a policy in the early period. However, there were some countries that had intentions to develop SHN policies in both period such as Ethiopia and Zambia.

Similar to Pillar 1, Pillar 2 also entails sizeable, fixed costs at the onset for infrastructure with lower costs in the following years to maintain the initial investment. All of the selected countries prioritized Pillar 2 in the early ESP, and a majority (80%) still prioritized these activities in the later period. Such activities included rehabilitating classrooms, building latrines and sanitation facilities, and accessing water supply to schools.

Establishing SHN education may be costly in terms of developing the resources and curriculum; however, over time the costs are more minimal. Thus in Figure 1, about 80% of countries addressed this pillar overall with most countries represented in the later period differing from the countries represented in the early phase. Only 3 of 25 countries did not have an ESP that reflected Pillar 3 in either phases. In many countries, the priority was on developed HIV education while skills-based health education more generally was less cited as a priority. In total, 12 countries prioritized life skills education. Ghana developed a curriculum for SHN while Rwanda prioritized funding for teacher training.

Unlike the other three pillars, activities under Pillar 4 typically require a sizeable, recurring annual budgetary allotment. In the early phase, 76% of countries provided or continued to deliver SHN services such as school meals and deworming. By the later phase, all countries were delivering SHN services. The increase from the earlier to the later phase highlights the universal presence of SHN gained over the 15-year period.

**Figure 2:**
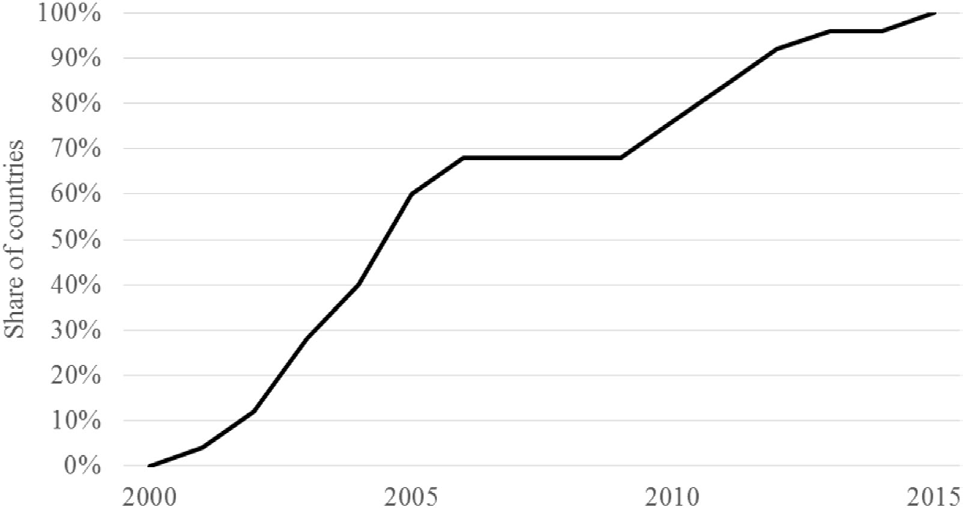
**Scale-up of school health and nutrition services in ESPs (FRESH Pillar 4)**. Note: sample includes 25 countries.

Figure 2 plots the cumulative distribution of the year in which country ESPs reflected Pillar 4. While no ESPs included Pillar 4 at the time of the World Education Forum, it was represented in ESPs in all countries by 2015. An increase to 70% was noted between 2000 and 2005, which was followed by a more gradual rise between 2010 and 2015.

**Figure 3:**
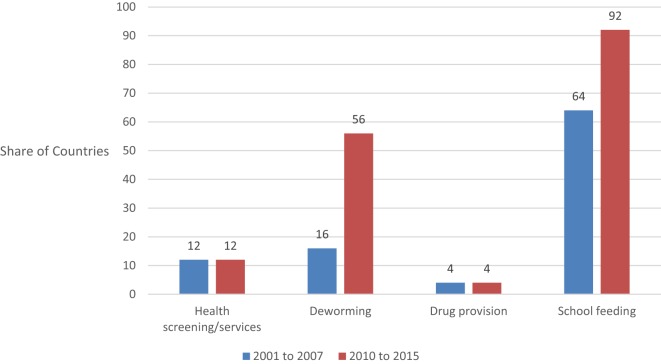
**ESPs inclusion of Pillar 4 by school health and nutrition intervention**.

Figure 3 presents the distribution of SHN activities included under Pillar 4. School feeding was the most common intervention followed by deworming, while health screening was a lower priority. Vision screening in particular has been identified as an important and cost-effective intervention, but is largely absent from the ESPs (1).

Both deworming and school feeding were increasingly prioritized and were the drivers behind the scale-up in SHN displayed in Figure 2.

**Figure 4:**
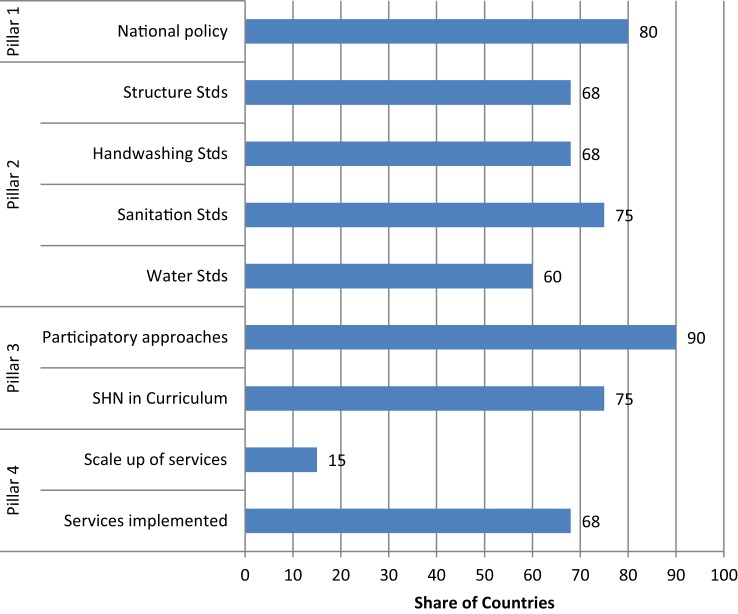
**Select findings from SABER analysis**. Note: sample is 16 countries.

While the ESP analysis presents needs as identified by governments, the findings from the SABER analysis illustrate the actual implementation of SHN in 16 countries. Figure 4 presents select findings classified by FRESH pillar. In terms of Pillar 1, 80% of countries reported having a national policy on school health and nutrition. These policies may not be a stand-alone, but memoranda of understanding of the inclusion of SHN in national policies. A majority of countries reported systems in place supporting Pillar 2, paralleling findings from the ESP analysis. In total, 60% of countries surveyed had water standards, while 75% had sanitation standards and 68% had sound school structures. The aforementioned standards may not be specific to schools but country general standards. In terms of facilities, the majority reported partial provision of water and sanitation facilities.

In regard to Pillar 3, 75% of the countries provided basic, accurate health, HIV, and nutrition and hygiene information in the school curriculum. Furthermore, more than 90% reported that participatory approaches were part of the curriculum and were used to teach key age-appropriate and gender-sensitive life skills for health themes. This may be a result of the efforts led by governments and partners to address HIV.

About two-thirds of countries reported having in place the SHN services identified in the situation analysis and outlined in the national policy (e.g., deworming, malaria control, school feeding, etc.). Ten of these countries had school-based screening programs and referrals to health systems. Less than 15%, however, reported a scale-up in the provision of these services.

The inclusion of a line in the education sector budget for SHN activities may support their sustainability and scale-up as has been noted for school feeding (10). In the SABER analysis, 12 of 16 countries had a budget line for SHN.

## Discussion

Since the World Education Forum was held in Dakar in 2000, SHN has been mainstreamed in the education sectors of sub-Saharan African countries. This analysis presents quantitative evidence from multiple countries, highlighting the scope of SHN as well as the adaptation to the country context. The ESP analysis highlights the increasing priority that governments place on SHN and its role in achieving education sector goals.

As education policies shifted from provision of quality education for the few to ensuring EFA during the 1990s, countries invested in building schools to accommodate the vast increase in enrollment (11). While the school health and nutrition component of ESPs typically focused on water, sanitation, and hygiene, some countries also sought to address violence and psychosocial issues under this pillar. These countries were typically in conflict at the time or recently post-conflict and included Liberia, Mozambique, and the Democratic Republic Congo. To provide an example, the objective in Liberia was “to make those provisions and arrangements that result in the school environment being clean, sanitary, violence-free and sufficiently conducive for all students, especially girls, to feel safe and at ease” and three activities were defined: (1) “provide a communication strategy to inform on the ‘children’s right’ charter and related issues”; (2) “develop and implement strategies that deal with sexual exploitation and violence in school issues ensuring the involvement of PTAs and the community”; and (3) “address safety and security of learners with disability in school issues” (12).

The scale-up in school feeding is likely to reflect heightened attention to its role as a social safety net following the 2007/2008 food and fuel price crises (9, 13). Recent estimates suggest that global investment in school feeding is up to US $75 billion and serves 368 million children (14). Similarly, the pattern in regards to deworming coincides with the global drug donation, which was initiated in 2012 (15). However, drug procurement policies, which are typically the domain for the health sector, remain at a very low level. Limited clarity regarding how the drug donation can be accessed as well as limited engagement between the education and health sectors may be factors limiting the scale-up in deworming activities over the period.

Governments also recognize the value represented in mainstreaming comprehensive, integrated SHN programing and seek more information on costs in order to better mainstream SHN into ESPs. In Ethiopia, for example, a cost analysis of the integration of school feeding with deworming and water, sanitation, and hygiene activities was undertaken to support budget planning. The analysis suggested a savings of 5–6% of total expenditure (16). Effective cross-sectoral policies and multi-sectoral steering committees can promote integrated, comprehensive programing.

While the focus of the present analysis is on ESPs, poverty reduction strategy papers (PRSPs) would also be a potentially useful tool for the integration and scale-up of SHN. PRSPs, developed in conjunction with development partners inclusive of the International Monetary Fund and the World Bank, act as overarching policy documents and define national priority areas for investment. However, to date, SHN as a cross-cutting issue has not been routinely included in PRSPs, a situation which would be worth rethinking.

Moving into the SDG era, SHN promises to help draw more children to school including the last 10%. SHN can address multiple needs for the most vulnerable children and provide the support needed to benefit from the education system. The evidence demonstrates that SHN not only can draw children to school but also help them learn and reach their potential (9).

A limitation to the current study is that ESPs do not report on program implementation or quality.^1^ This problem is exacerbated by the fact that many education statistical bulletins produced by governments in sub-Saharan Africa only partially report on school health activities, and that development partners investing in school health are often the primary source of SHN implementation data. The data reported here provide a useful overview of national priorities and what countries plan to achieve and also indicate key gaps in forward planning, but on-the-ground surveys are required to confirm the programmatic realities.

## Conclusion

The education sector has made remarkable progress during the MDG era, and the impact of national SHN programs during this time has had an impact on the health and educational outcomes of millions of school-aged children. Using schools as a platform to deliver multiple interventions has proven to be effective in contributing to education sector goals (17). As the world now pivots to address the multisectoral and social development contexts of the Strategic Development Goals the question is no longer whether school health and nutrition programs are necessary to meet the SDGs, but how to make the programs more scalable and sustainable moving toward 2030.

## Author Contributions

All the authors have provided substantial contributions to the development of the manuscript. BS, LD, BM, FP, MF, LB, and DB contributed to the overall conception and design. BS and AT gathered the data. BS, LD, BM, FP, MF, LB, DB, AT, KT, and AG analyzed the data. All the authors contributed to the interpretation of the data and the drafting of the manuscript, which was led by the authors affiliated with the Partnership for Child Development. All the authors have given final approval for the paper to be published in Frontiers and agree to be accountable for the content presented therein.

## Conflict of Interest Statement

The authors declare that the research was conducted in the absence of any commercial or financial relationships that could be construed as a potential conflict of interest. The reviewer EA and handling Editor declared their shared affiliation, and the handling Editor states that the process nevertheless met the standards of a fair and objective review.

## Footnotes

^1^Planipolis. Available from: http://www.iiep.unesco.org/fr/notre-expertise/planipolis.
